# Generation of an immortalized mesenchymal stem cell line producing a secreted biosensor protein for glucose monitoring

**DOI:** 10.1371/journal.pone.0185498

**Published:** 2017-09-26

**Authors:** Evangelia K. Siska, Itamar Weisman, Jacob Romano, Zoltán Ivics, Zsuzsanna Izsvák, Uriel Barkai, Spyros Petrakis, George Koliakos

**Affiliations:** 1 School of Medicine, Faculty of Life Sciences, Aristotle University of Thessaloniki, Thessaloniki, Greece; 2 Biohellenika SA Biotechnology Company, Thessaloniki, Greece; 3 GluSense Ltd, Rabin Science Parkm, Rehovot, Israel; 4 Paul-Ehrlich-Institute, Langen, Germany; 5 Max-Delbrück-Center for Molecular Medicine in the Helmholtz Association, Berlin, Germany; Augusta University, UNITED STATES

## Abstract

Diabetes is a chronic disease characterized by high levels of blood glucose. Diabetic patients should normalize these levels in order to avoid short and long term clinical complications. Presently, blood glucose monitoring is dependent on frequent finger pricking and enzyme based systems that analyze the drawn blood. Continuous blood glucose monitors are already on market but suffer from technical problems, inaccuracy and short operation time. A novel approach for continuous glucose monitoring is the development of implantable cell-based biosensors that emit light signals corresponding to glucose concentrations. Such devices use genetically modified cells expressing chimeric genes with glucose binding properties. MSCs are good candidates as carrier cells, as they can be genetically engineered and expanded into large numbers. They also possess immunomodulatory properties that, by reducing local inflammation, may assist long operation time.

Here, we generated a novel immortalized human MSC line co-expressing hTERT and a secreted glucose biosensor transgene using the *Sleeping Beauty* transposon technology. Genetically modified hMSCs retained their mesenchymal characteristics. Stable transgene expression was validated biochemically. Increased activity of hTERT was accompanied by elevated and constant level of stem cell pluripotency markers and subsequently, by MSC immortalization. Furthermore, these cells efficiently suppressed PBMC proliferation in MLR transwell assays, indicating that they possess immunomodulatory properties. Finally, biosensor protein produced by MSCs was used to quantify glucose in cell-free assays. Our results indicate that our immortalized MSCs are suitable for measuring glucose concentrations in a physiological range. Thus, they are appropriate for incorporation into a cell-based, immune-privileged, glucose-monitoring medical device.

## Introduction

During the past years diabetes has became a worldwide epidemic. It was ranked in the top ten leading causes of worldwide death cases in 2012 [1]. Global prevalence in 2014 was estimated at 8.5% and number of patients suffering from diabetes has risen from 108 million to 422 million between 1980 and 2014 [2] with a forecast of ever increasing numbers in future years.

Diabetes is dependent on either the absence or on the malfunction of a single pancreatic hormone, insulin. The disease is manifested by two related medical conditions, type I diabetes (T1D) and type II diabetes (T2D), the latter comprising between 85% and 90% of the affected population. In T2D, insulin signaling is affected while in T1D, pancreatic beta cells are destroyed by an autoimmune attack. During progression towards T1D, pancreatic islets are infiltrated by leukocytes consisting of CD4+ and CD8+ T cells, B cells, macrophages, plasma cells and dendritic cells [3–5].

Insulin deficiency causes an increase in blood glucose levels, which—in the long term—is the source of all diabetes complication, from nephropathy and retinopathy to neuropathy, cardiovascular diseases, etc. In T1D patients, blood glucose is controlled by regular injections of insulin. This is a demanding task as the dose of the injected hormone should be carefully calculated in order to avoid problematic hyperglycemia on the one hand but also life-threatening hypoglycemia episodes on the other hand.

Current methods for controlling blood glucose include a combination of frequent finger pricking used to measure glucose levels, followed by the administration of insulin at a pre-calculated dose. This procedure is repeated several times every day. Still, despite close attention, T1D patients experience risks of dangerously high and low blood-glucose levels. In order to make the insulin treatment safer, the amount of the insulin injected into the subcutis, and further acquired by the circulation, should precisely respond to differential changes in actual blood glucose concentrations. This concept drove the invention of continuous glucose monitoring systems (CGM), which allow for real-time reading of blood glucose levels.

Electrochemical CGM systems have been present on the market for more than 10 years. However, due to their insufficient accuracy, most of these commercial devices are cleared by the regulator for adjunctive use only. Presently, the only analytical method cleared by the regulator for indication of insulin dosing is finger pricking followed by enzyme-based glucose analysis (manual glucose meters). Recently, Dexcom (www.dexcom.com) received FDA committee recommendation to clear their 5th generation CGM for insulin dosing, and to 'eliminate the need for confirmatory fingersticks for daily glucose management decisions’ [6]. In addition, in order to maintain accuracy, currently available CGMs should be calibrated twice a day and replaced on a weekly basis. Despite these constrains, non-adjunctive CGM systems are expected to replace routine finger pricking [7] and be cost effective to the T1D community [8, 9].

Implantable CGM systems with improved accuracy and usable lifetime are presently being tested. First, an electrochemical system comprising glucose oxidase/catalase pair of enzymes, two complementary reading methods, independence from routine calibration and expected lifetime of over one year is being developed by GlySens Inc. (http://glysens.com/). Few attempts to develop fluorescent sensors were also executed during the past years. Among them, Biotex (http://www.biotexmedical.com/index.html) reported on developing a fiber-enclosed ConA-dextran FRET-based sensor [10–12]. However, the accuracy of the device (tested in rats and pigs) was moderate, and the development of an improved device was, apparently, ceased. The most advanced device in this category is the 'EverSense' CGM by Senseonics (http://www.senseonics.com/). It is a fluorescent, boronic acid based glucose-indicating polymer, expected to function for a period of about three months [13]. Overall accuracy of the sensor is similar to that obtained by standard electrochemical CGMs. However, it suffers from an accuracy Achilles heel when measuring hypoglycemic events where its precision is unsatisfactory [14].

A novel approach for creating a long term and accurate glucose measurement system is the use of engineered biosensor polypeptides. In this approach, a device containing an engineered cell type that is producing a transgenic biosensor could, theoretically, be implanted under the skin, where it may function for an indefinite period of time. To generate a chimeric glucose biosensor one could use bacterial periplasmic glucose/galactose receptor sensors. Notably, receptors from several bacterial species have been already cloned and their spatial organization defined (e.g. PDB 2HPH from *E*. *coli* or PDB 1GCA from *Salmonella typhimurium*). The *E*. *coli* Mglb has a natural dissociation constant of 0.21mM [15], which is inappropriate for measuring physiological concentrations of blood glucose in human. However, various mutants of this receptor with lower affinity values, in the millimolar range, render it suitable for being used as a physiological glucose meter [16–18].

In order to convert the mutated Mglb into a functional glucose biosensor, Förster resonance energy transfer (FRET) technology is being used. When two fluorescent proteins (e.g. Cyan Fluorescent Protein (CFP) and Yellow Fluorescent Protein (YFP)) are coupled with Mglb, a chimeric molecule is produced that have two conformational states. In the unbound state, a relaxed conformation places the two fluorescent polypeptide moieties at a distance that does not allow for FRET to occur. However, when glucose binds Mglb, conformational changes in the chimeric molecule brings the CFP and YFP fluorophores closer, thereby allowing FRET to occur. The intensity of the fluorescence emission is proportional to the ratio of bound to free biosensor molecules and, therefore, to glucose concentration.

Chimeric polypeptides consisting of Mglb and the two adjacent fluorescent proteins have been already designed and synthesized in several laboratories. A group led by Wolf Frommer generated a family of intracellular glucose sensors that was used to follow the dynamics of intracellular glucose and the activity of glucose transporters [19, 20]. Glucose affinity of the founding chimeric biosensor could be genetically manipulated, resulting in a family of related biosensors, characterized by dissociation constants in the range of 0.6 to 13.8mM [21]. Alternatively, Veetil et al. constructed a comparable biosensor comprising a red fluorescent acceptor protein (mCherry) with dissociation constant in a sub-millimolar range [22].

Cell-based biosensors usually use primary or immortalized cell lines as carriers [23, 24]. In order to measure intracellular glucose concentrations using FRET technology Fehr et al. have modified COS-7 cells [19]. However, monkey malignant cells are incompatible from being used in medical devices. On the other side, cell based biosensors based upon primary cells suffer from low lot to lot reproducibility and are, therefore, impractical for commercial use.

In this manuscript, we describe the generation of a human immortalized mesenchymal stem cell (MSC) line, stably transfected with a glucose biosensor transgene. The fluorescent polypeptides are secreted from the cells into a specific reservoir, where they may be used to measure glucose concentration present in biological fluids. Considering their immunomodulatory properties, MSCs may turn to be good candidates for implanted medical devices. In this paper, we explore a strategy of using engineered MSCs for continuous glucose monitoring.

## Material and methods

### Construction of transposon expression plasmids

A 63bp signal peptide [25] was synthesized and added at the N-terminus of a pcDNA3.1 FLII12Pglu-700uDelta6 plasmid, obtained from Addgene (https://www.addgene.org/) (plasmid #17866). Then, a 14-aa linker between the Mglb and citrine-YFP was deleted by site-directed mutagenesis, as previously described [20]. Finally, the Glucose Biosensor (GB) gene (consisting of a signal peptide, CFP, Mglb, -14aa linker, citrine-YFP) and its polyA tail were amplified by PCR using Phusion High-Fidelity polymerase (ThermoFischer, USA) and forward/reverse primers possessing the unique NheI and BglII site, respectively (S1 Table). Amplified DNA product was cloned into pJET1.2 vector using the CloneJet PCR Cloning Kit (ThermoFischer, USA). Its identity was verified by DNA sequencing. The EYFP-GW-polyA tail of a pT2-CAGGS-EYFP-GW [26] was replaced by the amplified Biosensor-polyA tail DNA fragment using the unique NheI-BglII sites, resulting in the generation of a pT2-CAGGS-Glucose Biosensor transposon expression plasmid. The nucleotide sequence of the final plasmid was verified by restriction digestion.

The SV40-neo^R^-polyA tail cassette was excised from a pT2-SV40-neo^R^ plasmid by HindIII and inserted into a pT2-CAGGS-EYFP-GW plasmid. The identity of the resulting pT2-SV40-neo^R^-CAGGS-EYFP-GW transposon expression plasmid was verified by restriction digestion and DNA sequencing. An entry clone encoding human telomerase reverse transcriptase (hTERT) (GeneID: 7015, clone ID: IOH36343) was shuttled into a pT2-SV40-neo^R^-CAGGS-EYFP-GW plasmid using LR clonase (ThermoFischer, USA), according to manufacturer’s instructions. The reaction mixture was used for transformation of Mach1 *E*. *coli* cells and transformants were selected on agar plates supplemented with 100μg/ml Ampicillin. The identity of pT2-neo^R^-hTERT plasmid was verified by BsrGI restriction digestion.

### Isolation of mesenchymal stem cells

MSCs were obtained from Biohellenika Biotechnology company. They were enzymatically isolated from an experimental sample of umbilical cord Wharton’s jelly under a consent form. Tissue pieces were digested with 2.7 mg/mL collagenase type I (ThermoFischer, USA) and 0.7 mg/mL hyaluronidase (Applichem, Germany) for 3h at 37°C, followed by incubation with 2.5% trypsin (Merck, Germany) for 30 min. Cell suspension was centrifuged at 500g for 30min at room temperature and pellet was resuspended in Dulbecco’s modified Eagle medium (DMEM) supplemented with 10% fetal bovine serum, penicillin (100 IU/mL) and streptomycin (100 μg/ mL). Isolated cells conformed with the minimal requirements of ISCT [27].

### Transfection

MSCs (2x10^5^ cells) were seeded in 6-well plates and transfected with 7.5 μg total plasmid DNA using xFect transfection reagent (Clontech, USA), according to manufacturer’s instructions. For generation of GB MSCs, cells were transfected with a mixture of SB100x transposase [28], pT2-CAGGS-Biosensor and pT2-SV40-neo^R^ transposon plasmids (1:8:1 ratio). Similarly, for generation of GB/hTERT MSCs cells were transfected with a mixture of SB100x transposase, pT2-CAGGS-Biosensor and pT2-SV40-neo^R^-hTERT transposon plasmids (1:8:1 ratio). Transfected cells were selected in culture medium containing 100μg/ml G418 and subcultured at a 1:2 ratio when they reached 95% confluence.

### Flow cytometry

Cells (1x10^6^) were stained with antibodies against hemopoietic (CD34, 45) or mesenchymal (CD29, 73, 90 and 105) stem cell markers. Unstained or MSCs stained with IgG isotype antibodies were used as negative controls. Cells were measured in a Cytomics FC500 flow cytometer (Beckman Coulter, USA) using the CXP2.2 software. Analysis of flow cytometry results was performed using the Flowing Software 2.5.1.

### Multilineage differentiation

MSCs were grown to 90% confluency and cultured for 28 days in StemPro Adipogenesis or Osteogenesis differentiation medium (Thermo Fisher, USA). Adipogenic and osteogenic terminal differentiation was monitored by Oil Red O (Sigma Aldrich, USA) or Alizarine Red S (Sigma Aldrich, USA) staining, respectively.

### RT-qPCR

Total RNA was extracted from MSCs using the NucleoSpin RNA kit (Macherey-Nagel, Germany) according to manufacturer’s instructions. Specific primers for Oct-4, Nanog and Sox-2 genes were designed using the Primer-Blast program. Their sequences are shown in S1 Table. cDNA generation and RT-qPCR reactions were performed using the KAPA SYBR Fast 1-step kit (Kapa Biosystems, USA) in a Rotor-Gene 6000 operating system. Each experiment was performed in triplicate. Amplified RT-qPCR products were analyzed in a 2% agarose gel electrophoresis.

### Microscopy

Cells were observed in an inverted AxioVert CFL40 Zeiss microscope equipped with an HBO 50 mercury lamp and reflectors, under brightfield or fluorescence filter sets for YFP (excitation 488 nm, emission 517 nm). Image acquisition was performed using the Fluorescence Lite software module of AxioVision LE (Carl Zeiss, Germany).

### Western blot

MSCs (5x10^5^ cells) were lysed in 200ul 1% SDS PBS. Cell extracts and supernatants were analyzed in SDS-PAGE electrophoresis followed by electrotransfer onto nitrocellulose membrane (Macherey-Nagel, Germany) using a semidry apparatus. Membranes were blocked with 5% w/v skim milk powder (Sigma Aldrich, USA) in PBST for 1h and incubated with mouse anti-GFP mAb (Millipore, MAB2510, 1:2000 dilution) or rabbit anti-TERT (Novus Biologicals, USA, NB120-32020, 1:2000 dilution) antibodies overnight. For loading controls, membranes were blotted with rabbit anti-GAPDH (Santa Cruz, USA, sc-25778, 1:1000 dilution) antibody. After incubation with the appropriate secondary alkaline phosphatase-conjugated antibody (Merck, Germany), membranes were developed with AP substrate NBT/BCIP (Applichem, Germany).

### Telomerase activity

Telomerase activity was determined using the TeloTAGGG Telomerase PCR Elisa Kit (Roche, Switzerland). MSCs (2x10^5^ cells) were lysed in 200ul Lysis buffer and used for the telomeric repeat amplification protocol (TRAP) with PCR. Telomerase activity was described in a semi-quantitative manner as “relative telomerase activity” (RTA), compared to a control template sample with a known number of telomeric repeats.

### Soft agar assay

For soft agar assays, 6x10^4^ MSCs or HEK293T cells were mixed with complete culture medium containing 0.7% agar (DIFCO, USA) and poured onto a layer of 1% agarose DMEM (Applichem, Germany) in 10cm tissue culture plates. Cells were cultured for 21 days.

### Mixed lymphocyte reaction

Human peripheral blood monocyte cells (PBMCs) from a healthy donor were isolated on Histopaque (1.077 g/ml, Sigma Aldrich, USA) density gradient centrifugation and resuspended in DMEM supplemented with 10% FBS, penicillin (100 IU/mL) and streptomycin (100μg/ml). For mixed lymphocyte reactions (MLR), 0.4μM pore size transwells (Greiner Bio-One, Germany) were used. To induce T cell proliferation, transwell plates were coated with anti-CD3 antibody (5μg/ml, AbD Serotec, UK). Then, PBMCs (2.5x10^6^) were resuspended in 500μl culture medium containing anti-CD28 antibody (1μg/ml, AbD Serotec, UK) and IL-2 (5U, Sino Biological, China) and added in the lower chamber. Control experiments were performed in the absence of stimulants. MSCs (0.5x10^6^) were resuspended in 200μl culture medium and placed onto the upper chamber of transwells. Cells were cultured for three days. Then, cell viability and total cell number or PBMCs in the lower chamber was measured using the Cyto Tox-Glo cytotoxicity kit (Promega, USA), according to manufacturer’s instructions.

### Fluorescence measurements

Control or GB/hTERT MSCs were cultured in phenol-red free Dulbecco’s modified Eagle medium (DMEM) supplemented with 10% fetal bovine serum, penicillin (100 IU/mL) and streptomycin (100μg/mL). Then, supernatants were collected and YFP fluorescent intensity correlating to secreted GB protein was measured in a Victor3 plate reader (PerkinElmer, USA) equipped with the appropriate filters. For fluorescence spectral scans, samples were measured in emission range from 450 to 690nm with 2nm intervals.

### FRET assay

For estimation of FRET signal, cells were cultured in phenol-red free low glucose DMEM/F12 (Sigma Aldrich, USA) supplemented with 2% fetal bovine serum, penicillin (100 IU/mL) and streptomycin (100 μg/ mL) for 72 hrs. Culture medium containing secreted biosensor was collected and concentrated using Vivaspin 6 50K centrifugal filters (Sartorius, Germany). The media was exchanged three times with 50mM Tris-HCl pH 7.6, 100mM NaCl, 1mM CaCl_2_ buffer to reduce glucose concentration to <0.1mM. Samples were then mixed with increasing concentrations of glucose solutions (0.01-100mM final glucose concentration). Fluorescence was measured using ZEISS Observer D1 microscope system and the following excitation/emission filter sets: CFP 436nm/480nm, FRET 436nm/530nm at 25°C. FRET signal was calculated as the ratio of:

$$\;\frac{FRET\;436nm/530nm_{\left(GB+glucose\right)}}{CFP\;436nm/480nm_{\left(GB+glucose\right)}}$$

### Statistical analysis and densitometry

Statistical analysis of the results (p value calculation) was performed using the GraphPad prism software. Data from three different experiments are presented as mean ± SD. Quantification of proteins in each sample was performed using the ImageJ software.

## Results

### Characterization of control MSCs

Wharton’s jelly MSCs are stem cells present in several adult tissues and have a good proliferative potential. First, plastic-adherent human MSCs with a fibroblastic morphology were isolated and expanded *ex vivo* (S1A Fig). Flow cytometry analysis verified their mesenchymal origin, as they were negative for the hematopoietic stem cell markers CD34 and CD45, but expressed high levels of the mesenchymal stem cell markers CD29, CD73, CD90 and CD105 (S1B Fig). The expanded cells also retained their differentiation potential towards the osteocytic and adipocytic lineages. Differentiated cells were positively stained with Alizarin Red, which detects calcium deposits of functional osteocytes and with Oil Red O, indicating cytoplasmic lipid droplets of adipocytes, respectively (S1C Fig).

### Generation of MSCs stably expressing glucose biosensor gene

**Fig 1:**
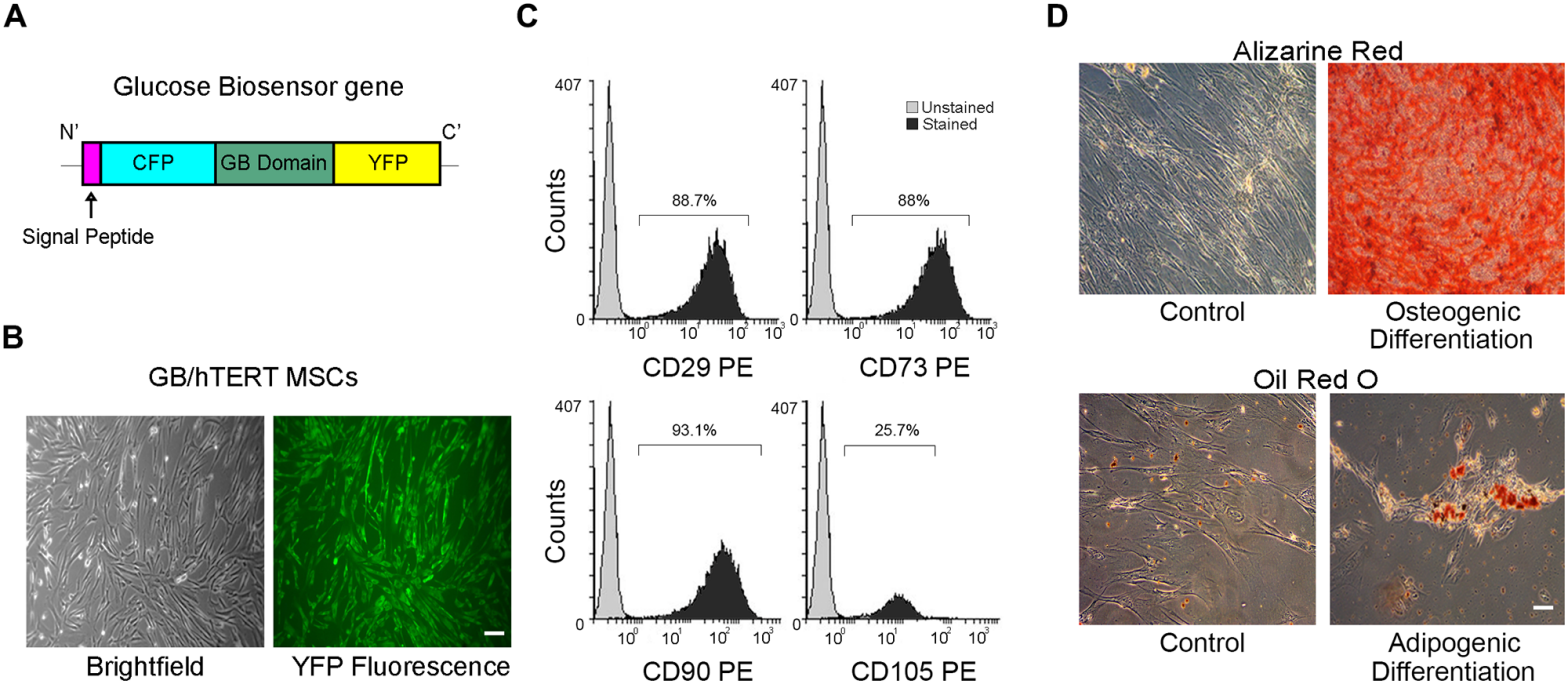
Characterization of GB/hTERT MSCs. (A) Schematic representation of a Glucose Biosensor (GB) gene used for the genetic modification of MSCs. The gene encodes a chimeric protein consisting of a signal peptide allowing protein secretion followed by Cyan Fluorescent Protein (CFP), a glucose binding domain and Yellow Fluorescent Protein (YFP). (B) Optical and YFP fluorescence microscopy of GB/hTERT MSCs (scale bar: 100μm). (C) Flow cytometry of GB/hTERT MSCs for mesenchymal CD29, 73, 90, 105 markers. Unstained cells were used as control. (D) Staining of control or differentiated GB/hTERT cells with Alizarine Red/Oil Red O indicative of osteogenic/adipogenic differentiation, respectively (scale bar: 100μm).

MSCs at passage 5 were genetically modified as to express a previously described glucose biosensor (GB) transgene [20] using the *Sleeping Beauty* transposon technology. This synthetic gene encodes a fusion protein consisting of an N’-terminal signal peptide, Cyan Fluorescent Protein (CFP), a glucose binding domain and Yellow Fluorescent Protein (YFP) (Fig 1A). Upon glucose binding to its binding domain, GB protein releases a measurable FRET signal, which is proportional to glucose concentration. GB MSCs were tested whether they retain their mesenchymal properties. After G418 selection, cells with a fibroblastic morphology homogeneously expressed the GB gene as indicated by fluorescence microscopy (S2A Fig). GB MSCs also expressed most mesenchymal stem cell markers at high levels (S2B Fig), and retained their differentiation potential towards osteocytes and adipocytes (S2C Fig).

MSCs are known to undergo replicative senescence upon *ex vivo* expansion; this phenomenon reduces their productive lifespan, their usability in some cell therapy approaches and alters their mesenchymal properties. To overcome this limitation, we generated a cell line co-expressing both the GB transgene and the hTERT gene. GB/hTERT MSCs displayed fibroblastic morphology, expressed mesenchymal stem cell markers (CD29, CD73 and CD90), and were homogeneously fluorescent, indicating that they properly express the GB transgene (Fig 1B). The cells also expressed mesenchymal stem cell markers (CD29, CD73 and CD90) (Fig 1C). Interestingly, compared with control MSCs, only 25.7% of genetically modified cells expressed the CD105 antigen. Multilineage differentiation potential of GB/hTERT MSCs was also tested. Differentiated cells were efficiently stained with Alizarin Red or Oil Red O, confirming that genetically modified cells retain their mesenchymal properties (Fig 1D).

**Fig 2:**
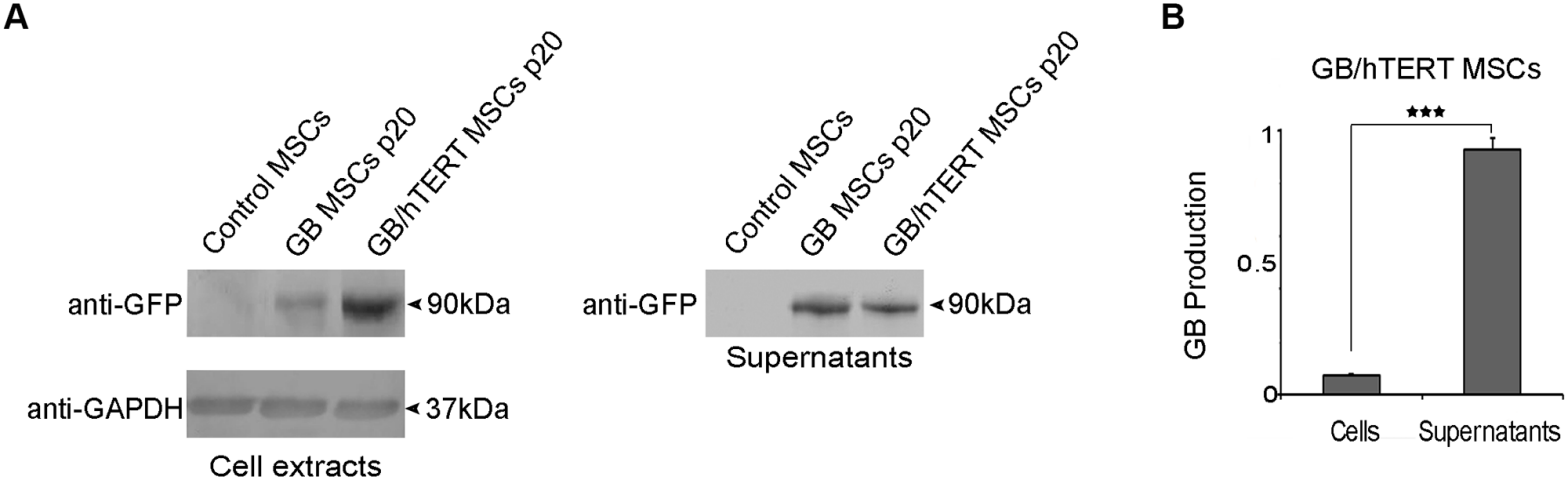
GB production in genetically modified MSCs. (A) Immunoblots for GB protein in control, GB and GB/hTERT MSC extract and cell culture supernatants (12.5% and 2.5% of total, respectively) using an anti-GFP antibody. GAPDH was used as a loading control. (B) Densitometry quantification of GB production in GB/hTERT cells and secretion in cell culture supernatant. Data from 3 independent experiments are presented as mean ± SD (***: p<0.001).

Next, we tested the expression of GB protein in GB- and GB/hTERT MSCs. Extracts from genetically modified cells at passage 20 (1/8 of total cell extract) were analyzed by SDS-PAGE. While immunoblotting with an anti-GFP antibody detected a band at 90kDa, no such band was detected in non-engineered, control MSCs, confirming that both GB and GB/hTERT MSCs properly express the GB protein. Supernatants from these cell cultures (1/40 of total cell culture supernatant) were also probed for the GB protein. A band at 90kDa (the expected molecular mass of GB) was detected in samples from genetically modified cells, but not in samples from control MSCs (Fig 2A). This indicates that GB and GB/hTERT MSCs secrete the GB protein in cell culture supernatant. Finally, the intensity of the bands corresponding to the GB protein was measured in both cell extracts and cell culture supernatants. Protein amounts were estimated by densitometry. Data analysis indicated that around 95% of the total GB protein produced by GB/hTERT MSCs is secreted into the supernatant (Fig 2B).

### Expression of hTERT in GB and GB/hTERT MSCs

**Fig 3:**
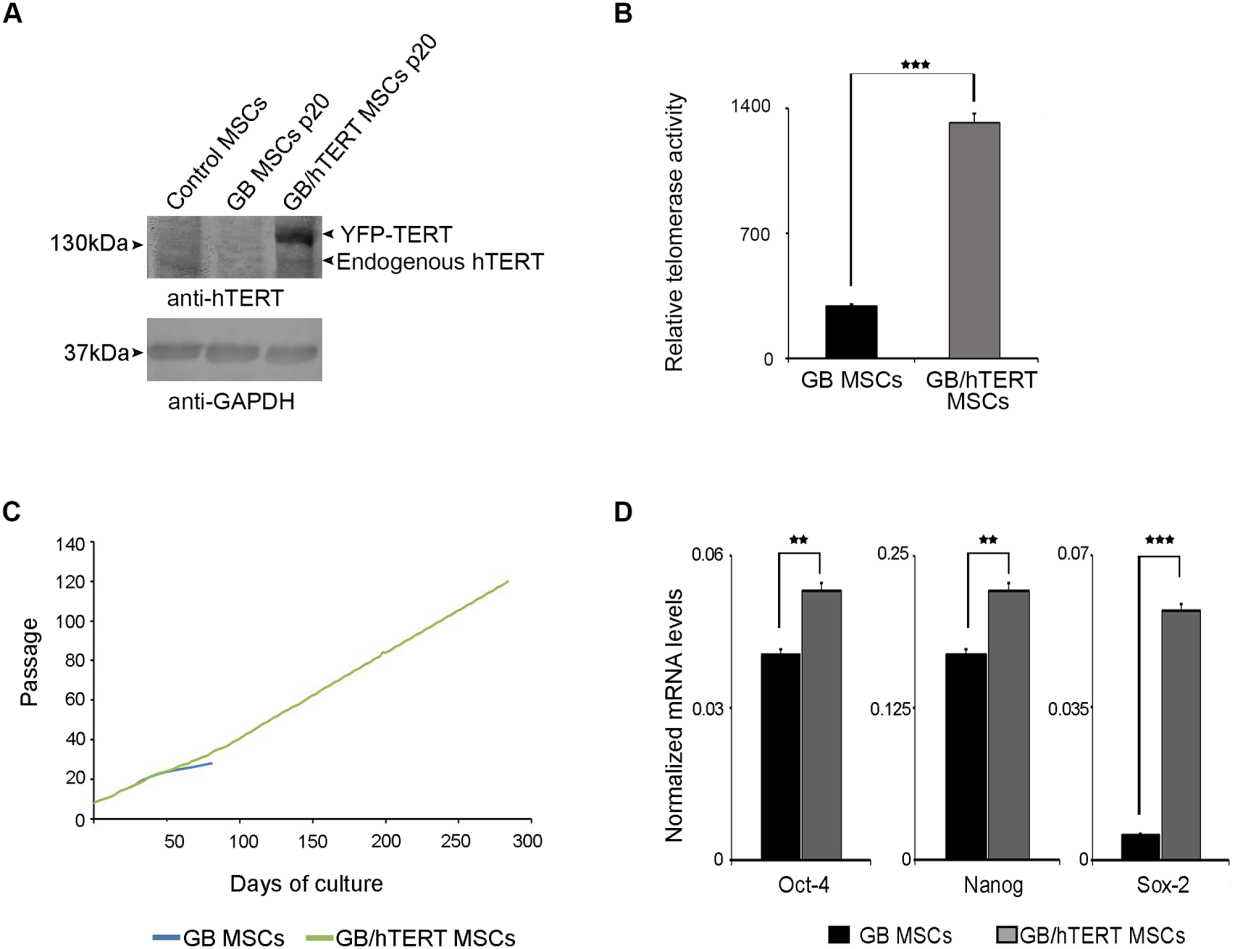
Self-renewal potential of genetically modified MSCs. (A) Immunoblots for hTERT in control, GB and GB/hTERT MSC extract using an anti-hTERT antibody. GAPDH was used as a loading control (B) Relative telomerase activity in GB and GB/hTERT MSCs at passage 20. (C) Cumulative population doublings of GB and GB/hTERT MSCs over time. GB MSCs reached replicative senescence at passage 28. (D) mRNA levels of Oct-4, Sox-2 and Nanog pluripotency markers in GB and GB/hTERT MSCs (***: p<0.001, **: p<0.01). Results in (B) and (D) are shown as mean ± SD of 3 independent experiments.

Next, we investigated the expression of hTERT in genetically modified MSCs. Cell extracts were analyzed by SDS-PAGE, and probed with an anti-hTERT antibody. Immunoblotting detected a 127 kDa band (the expected molecular mass of endogenously expressed hTERT) in samples from control, GB or GB/hTERT MSCs, corresponding to endogenous hTERT. As expected, densitometry analysis indicated that both control and GB MSCs express significantly lower levels of endogenous TERT at passage 15 and 20, respectively, compared to GB/hTERT MSCs at passage number 20 (S3 Fig). Additionally, an extra band at 147 kDa was also detected in GB/hTERT MSCs. This high intensity band corresponds to ectopically expressed YFP-hTERT, and confirms that GB/hTERT MSCs express high levels of the hTERT transgene (Fig 3A). Next we asked whether higher hTERT expression in GB/hTERT MSCs is associated with correspondingly higher telomerase activity. Relative telomerase activity was estimated in GB or GB/hTERT cells at passage 20 using an assay which allows quantitative determination of telomerase levels. Our analysis showed that GB/hTERT MSCs have a 4.5-fold increase in telomerase activity compared to GB MSCs at a similar passage (Fig 3B). Taken together, these results suggest that GB/hTERT MSCs produce high levels of a functional hTERT protein.

The maintenance of telomerase activity in MSCs potentially protects them from senescence. To test this assumption, we maintained long-term cultures of genetically modified MSCs and calculated their growth curves. GB MSCs were cultured up to passage 28. At this time point, cells increased in size, were growth-arrested and eventually died in culture. These observations are in agreement with the previous result that GB MSCs at passage 20 express only low levels of endogenous hTERT (Fig 3A) that cannot support their long-term proliferation potential. In contrast, GB/hTERT MSCs were maintained in culture for over 300 days, and cultured up to 127 passages with a population doubling time of 48-72hrs (Fig 3C). This indicates that ectopic expression of YFP-hTERT preserves the proliferation potential of GB/hTERT MSCs and protects these cells from senescence.

The self-renewal capacity of adult stem cells correlates with the expression of pluripotency markers Oct-4, Sox-2 and Nanog. To determine whether the proliferative capacity of GB/hTERT MSCs is associated with the high expression of these markers, we estimated their transcription levels by RT-qPCR. The identity of the amplified fragments in RT-qPCR was validated by melting curve analysis and agarose gel electrophoresis (S4 Fig). Our analysis showed that GB/hTERT MSCs have significantly higher mRNA levels of pluripotency markers than GB MSCs at a similar passage. More specifically, GB/hTERT MSCs expressed 31% and 86% higher mRNA levels of Oct-4 and Sox-2, respectively. Interestingly, GB/hTERT MSCs had a 10-fold increase in Nanog mRNA levels (Fig 3D). These results suggest that the higher proliferative capacity of GB/hTERT versus GB MSCs is associated with the higher expression levels of pluripotency stem cell mediators.

In principle, immortalized MSCs may spontaneously transform into cancer stem cells. We therefore evaluated the tumorigenic potential of immortalized GB/hTERT MSCs. Cells at passage 85 were negative for the expression of CD133, a well-known cancer cell marker which is ubiquitously expressed in e.g. lung cancer A549 cells (S5A Fig). Additionally, GB/hTERT MSCs at passage 85 were tested for their potential to form colonies in soft-agar assays, indicative of cellular transformation. Neither GB/hTERT at passage 85 nor control MSCs at passage 15 formed colonies in contrast to positive control HEK293 cells (S5B Fig). Taken together, these results suggest that overexpression of hTERT in GB/hTERT MSCs and their subsequent immortalization do not transform them into cancer stem cells; they also indicate that GB/hTERT MSCs might be safe for biomedical applications.

### GB/hTERT MSCs suppress the proliferation of lymphocytes in vitro

MSCs have the ability to suppress the proliferation of lymphocytes in a paracrine manner. Cells with such properties express high levels of CD200, CD274 and CD276 markers. Unmodified MSCs used in our study expressed high levels of these markers, suggesting that they possess certain immunomodulatory properties (S6 Fig). Next, we asked whether GB/hTERT MSCs are still capable of suppressing the proliferation of lymphocytes. To this end, we first set up PBMC proliferation assays. 78.89% of isolated PBMCs were positive for the CD3 T cell marker as measured by flow cytometry (S7C Fig), indicating that this fraction is highly enriched in T cells. Then, PBMCs were stimulated with mitogens and their proliferation was quantified. Cells efficiently proliferated forming visible rods in cell culture (S7A Fig). Stimulation of PBMCs resulted in a 52% increase in the total cell number as indicated by cell-titration assays (S7B Fig). Flow cytometry indicated that the fraction of stimulated PBMCs was still highly enriched in T cells; 86.03% of all cells were positive for CD3. Additionally, there was a 2.8-fold increase in the absolute number of CD3^+^ cells (S7C Fig), suggesting that T cells efficiently proliferated under these conditions.

**Fig 4:**
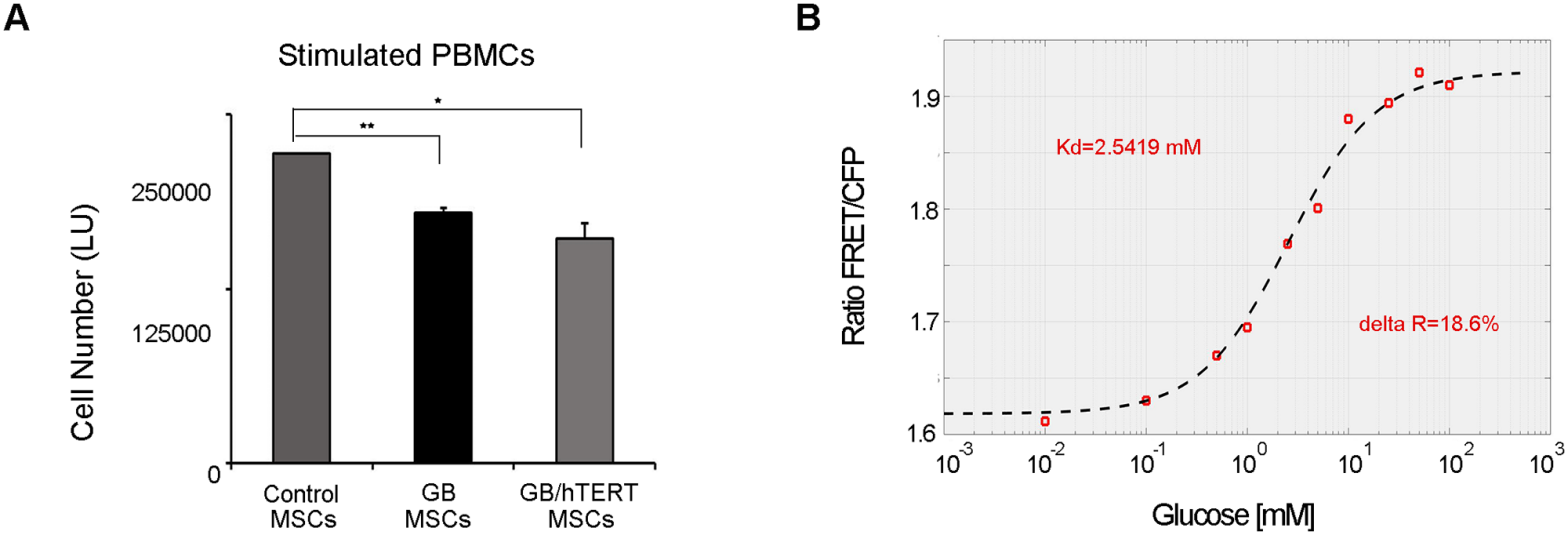
**A. Immunosuppressive properties of genetically modified MSCs**. Transwell co-cultures of stimulated PBMCs with GB or GB/hTERT MSCs. Cultures with DMEM were used as control. Total cell number in lower chamber containing stimulated PBMCs is expressed as luminescence units (**: p<0.01, *: p<0.05). **B. Detection of a glucose-dependent FRET signal using GB protein**. FRET signal detected in a GB/hTERT MSC culture supernatant and mixed with increasing glucose concentrations (0.01-100mM). Signal corresponds to a proportional GB protein/glucose complex depicted in a kinetic curve with Kd value of 2.5419mM and delta R of 18.6% at 24°C.

In order to quantify the effect of MSCs on PBMC proliferation, we performed MLR assays using transwell plates. The design of these plates allows for chemical communication between MSC-containing upper chamber and PBMC-loaded lower chamber but prevents migration and physical mixing of the different cell populations. Cell-titration assays in the lower chamber containing stimulated PBMCs indicated that GB/hTERT MSCs significantly suppressed PBMC and subsequently T cell proliferation (32% reduction in total cell number compared to control/stimulated PBMCs co-cultured with DMEM). A similar result was obtained with GB MSCs (28% reduction in total cell number compared to control/stimulated PBMCs co-cultured with DMEM) (Fig 4A). We also quantified the number of dead cells in the lower chamber. Interestingly, we found an almost double amount of dead cells in co-culture experiments of stimulated PBMCs with GB or GB/hTERT MSCs. This indicates that secreted cytokines from genetically modified MSCs induce cell death mechanisms in PBMCs *in vitro* (S8 Fig). Taken together, these results indicate that GB/hTERT MSCs have an immunosuppressive effect *in vitro* and retain their immunomodulatory properties.

### Estimation of glucose concentration in vitro using GB protein

GB/hTERT MSCs produce high levels of secreted GB protein, which could be used to quantify glucose concentration in a biological sample by FRET measurements. To do so, we first measured GB protein secretion in cell culture supernatants using a fluorescence plate reader. We detected an increasing YFP signal within 72hrs corresponding to the increasing amounts of the secreted GB protein. No such signal was detected in supernatants from control MSC cultures (S9A Fig). Then, cells were cultured in glucose-free medium; supernatants containing GB protein were collected, and mixed with a 25mM glucose solution. Upon glucose addition, a measurable shift in the basal FRET signal was detected (S9B Fig), indicating the formation of a glucose-GB protein complex.

Then, GB protein was mixed with increasing concentrations of glucose (0-55mM) and fluorescence spectral scan analysis was performed after CFP excitation at 436nm. A FRET signal was detected with a peak at 530nm corresponding to YFP fluorescence. Fluorescence intensity increased at higher glucose concentrations and reached a plateau at 20mM glucose in solution. No such signal was observed in supernatants from control MSCs (S9C Fig). Finally, a quantitative FRET assay was developed for the estimation of glucose concentration in solution. GB protein was mixed with increasing concentrations of glucose (0.01-100mM). FRET signal corresponding to a glucose-GB protein complex was calculated after subtraction of a background CFP signal. Dose-dependent analysis showed that increasing glucose concentrations correlate with a proportional FRET signal in solvents. The resulting kinetic curve had a Kd value of 2.5419mM at 24°C (Fig 4B). This assay may efficiently detect glucose concentrations between 1-10mM *in vitro* which is within the range of blood glucose levels, while the FRET signal becomes saturated at higher concentrations. Taken together, these results indicate that GB/hTERT MSCs produce and secrete a functional glucose biosensor protein, which may be used to measure glucose concentrations in biological fluids.

## Discussion

In the present study, we describe the generation and properties of an immortalized human MSC line that can be used to measure extracellular glucose levels. To do so, we stably transfected Wharton’s Jelly hMSCs with both hTERT and a FRET-based biosensor gene which permits glucose measurements in a physiological range [20]. We used the *Sleeping Beauty* system that is highly suitable for stable expression of a transgene in various stem cells [29–31].

FRET technology offers several advantages. Response time is fast and proper design allows for high sensitivity to a specific ligand. It also provides both proximity and kinetic information. Therefore, it is widely used to identify and also quantify protein-protein interactions [32]. Likewise, FRET-based biosensors have been successfully used to quantify activity of intracellular kinases and their response to drugs in living cells [33]. Thus, such biosensors are valuable tools with potential applications in the field of personalized medicine.

A signal peptide was attached at the N-terminus of the GB gene, allowing the recombinant protein to be secreted. Essentially, our results demonstrated that the majority of the expressed GB was properly secreted and was detected in the cell culture supernatant (Fig 2). Also, the secreted biosensor protein was functional. When combined with glucose it gave a measurable FRET signal, proportional to the concentration of glucose (Fig 4B and S9C Fig). These results collectively indicate that our reporter is suitable for quantitative estimation of glucose concentration in cell-free assays.

Wharton’s Jelly MSCs were selected for the production of the recombinant GB protein. This decision was based on several reasons. Firstly, MSCs are primary cells that do not carry any genetic modification and can be easily expanded to high numbers. Secondly, MSCs are hypoimmunogenic. They do not express HLA class II antigens [34] or the co-stimulatory molecules CD40, CD80 and CD86. On the other hand, they differentially express various HLA class I antigen subtypes. While HLA-G, induces Treg cell proliferation [35] that protects them against NK cells [36], expression of HLA-B is low thus, protecting the cells from complement-dependent cytotoxicity [37]. MSCs also produce molecular mediators like IDO, TGF-β and PGE2 that suppress T cell proliferation [38–40]. These cells are, thus, ideal for transplantation or immunomodulation in *in vivo* applications.

Our results showed that MSCs retain their immunomodulatory characteristics after being genetically modified with GB gene. The transgenic cells are plastic-adherent, have fibroblastic morphology, can differentiate into mesodermal cell lineages and express mesenchymal stem cell CD surface markers (Fig 1 and S2 Fig). Interestingly, we observed that genetically modified MSCs express low levels of CD105, an auxiliary component of the TGF-β receptor complex. CD105 is involved in cell proliferation, differentiation and migration [41]. Its expression is affected by passage number and cell confluency in culture. CD105^-^ and CD105^+^ MSCs were shown to have similar properties (growth potential and CD marker expression) but varied in their differentiation potential. CD105^-^ MSCs define a subpopulation of cells characterized by increased immunosuppressive capabilities [42] rendering them more appropriate for our applications. However, we cannot exclude the possibility that expression of CD105 in GB and GB/hTERT MSCs is affected by their genetic modification.

MSCs when cultured are subjected to replicative senescence, a phenomenon known as the Hayflick limit. This limit indicates that cells may divide for a specific number of passages, but then enter into a phase in which they can no longer proliferate. Replicative senescence is accompanied by loss of their differentiation ability, morphological and biochemical changes [43].

One of the most important changes in cells entering senescence is the shortening of telomeres with each cell division, resulting from gradual loss of hTERT activity. In our experiments, replicative senescence was more intense in GB MSCs as these cells stopped proliferating at passage 28 (Fig 3C), and showed reduced expression of endogenous hTERT compared to their control counterparts (Fig 3A and S3 Fig). However, co-expression of GB and hTERT restored the proliferative potential of MSCs. This observation was accompanied with the relatively constant expression levels of pluripotency markers Oct-4, Sox-2 and Nanog (Fig 3D). We recently showed that Oct-4 and Nanog regulate the expression of genes associated with the replicative senescence of MSCs [44]. Thus, increased levels of these markers in GB/hTERT MSCs may upregulate genes with anti-aging activity contributing to the preservation of MSC stemness. GB/hTERT MSCs express and secrete a functional GB protein (Fig 2) that may measure glucose concentrations in solutions *in vitro* (Fig 4B and S9C Fig). Given their high proliferative capacity, supernatants from GB/hTERT MSC cultures can be used for the purification of large amounts of GB protein appropriate for the measurement of glucose in high-throughput cell-free assays.

In contrast to strategies using primary cells that suffer from low reproducibility, and are therefore not practical for commercial use, we propose that GB/hTERT MSCs can also be incorporated in cell-based biosensors. The good proliferation rate of GB/hTERT MSCs allows for the construction of large biobanks with cells of identical characteristics. Although immortalization of GB/hTERT MSCs may also raise biosafety concerns, we show that these cells, by contrast to certain common laboratory cell lines [45], do not form colonies in soft agar assays. Thus, they do not spontaneously transform into cancer cells (S5B Fig), indicating that GB/hTERT MSCs might be suitable for use in biomedical applications from a biosafety point of view.

Besides their stem cell properties, MSCs possess the ability to impart profound immunomodulatory effects which may be due to the presence of cytokines secreted in conditioned medium. Nevertheless, these immunomodulatory roles have been recently assigned to MSCs [46], and markers describing this lineage were established [47]. MSCs have been already tested in small animal models to treat diseases where immunomodulation is thought to be the main operative mechanism [48]. They have been reported to suppress T cell proliferation in a variety of assays using stimuli including mitogens, CD3/CD28 and alloantigens. Although the exact mechanism remains largely unknown, suppression occurs independently of the MHC complex, and appears to be mediated by secreted cytokines, such as TGFβ-1, hepatocyte growth factors [49–51] and factors like PGE-2 and NO [52]. Importantly, our GB/hTERT MSCs retained their immunomodulatory properties, and efficiently suppressed T cell proliferation in MLR assays (Fig 4A). Transplantation devices, even if partially protected from the host immune system by a semi-permeable membrane, induce immune response, resulting in a decreased efficacy of the system. Although anti-inflammatory drugs such as steroids and NSAID could extend the productive life span of the device, long term use of these drugs is not recommended. In principle, transplantation of GB/hTERT cells *in vivo* may generate a local immunosuppressive microenvironment for the cell-based glucose biosensor. A cell-based glucose biosensor containing GB/hTERT MSCs may measure glucose levels at the physiological range and in parallel create an immune privileged implantation site, extending the life span of the biosensor.

## Supporting information

S1 Fig Characterization of control MSCs. (A) Optical microscopy of control MSCs (scale bar: 100μm). (B) Flow cytometry for hemopoietic CD34, 45 or mesenchymal CD29, 73, 90, 105 markers. (C) Staining of control or differentiated cells with Alizarine Red/Oil Red O indicative of osteogenic/adipogenic differentiation, respectively (scale bar: 100μm).
(TIF)

S2 Fig Characterization of GB MSCs. (A) Optical and fluorescence microscopy of GB MSCs (scale bar: 100μm). (B) Flow cytometry for mesenchymal CD29, 73, 90, 105 markers. (C) Staining of control or differentiated cells with Alizarine Red/Oil Red O indicative of osteogenic/adipogenic differentiation, respectively (scale bar: 100μm).
(TIF)

S3 Fig Endogenous hTERT levels in control and genetically modified MSCs. Protein levels of endogenous hTERT (Fig 3A) were quantified in control (passage 15), GB or GB/hTERT MSCs (passage 20) using the ImageJ software. GAPDH protein levels were used as loading controls.
(TIF)

S4 Fig Real time PCR. Amplified fragments of Oct-4, Nanog and Sox-2 mRNA in RT-qPCR reactions.
(TIF)

S5 Fig Tumorigenicity assays. (A) Flow cytometry of GB/hTERT MSCs and lung cancer A549 cells for the cancer cell marker CD133. (B) Soft agar assays of GB/hTERT MSCs at passage 85. HEK293T cells and control MSCs at passage 15 were used as positive and negative control, respectively (scale bar: 50μm).
(TIF)

S6 Fig Expression of immunomodulatory markers. Flow cytometry of control MSCs for the immunomodulatory cell markers CD200, 276 and 274.
(TIF)

S7 Fig Proliferation of stimulated PBMCs. (A) Optical microscopy of unstimulated and stimulated PBMCs (scale bar: 100μm). (B) Measurement of cell proliferation. Luminescence units (LU) correspond to total cell number. Data from 3 independent experiments are presented as mean ± SD (***: p<0.001). (C) Flow cytometry of unstimulated and stimulated PBMCs for CD3 T cell marker.
(TIF)

S8 Fig Quantification of PBMC death in MLR assays. Cell death of stimulated PBMCs co-cultured with GB or GB/hTERT MSCs in transwell plates is expressed as a percentage (%) of total cell number. Control experiments were performed in the absence of MSCs in MLR assays. Data from 3 independent experiments are presented as mean ± SD (**: p<0.01, *: p<0.05).
(TIF)

S9 Fig (A) Quantification of GB secretion by GB/hTERT MSCs as indicated by YFP measurement in cell culture supernatant (24-72h cultures). Control measurements were performed in samples from control MSC culture. (B) Detection of FRET signal in GB/hTERT cell culture supernatant in the absence or presence of glucose (25mM). Data from 3 independent experiments are presented as mean ± SD (***: p<0.001). (C) Fluorescence spectral scan analysis and detection of FRET signal in GB/hTERT cell culture supernatant mixed with various glucose concentrations (0-55mM) (RFU: Relative Fluorescence Units).
(TIF)

S10 Fig Original immunoblots presented in (A) Fig 2A and (B) Fig 3A. (TIF)

S1 Table Primer sequences. Sequences of primers used for the amplification of GB gene or fragments of target genes in RT-qPCR reactions.
(XLSX)

S2 Table Individual data points of bar and curve graphs presented in main and supplementary figures of the manuscript. (XLS)

## Abbreviations

CGM: Continuous Glucose Monitoring
CFP: Cyan Fluorescent Protein
FRET: Fluorescence Resonance Energy Transfer
GB: Glucose Biosensor
hTERT: human Telomerase Reverse Transcriptase
MLR: Mixed Lymphocyte Reactions
MSCs: Mesenchymal Stem Cells
PBMCs: Human Peripheral Blood Monocyte Cells
T1D: Type I diabetes
T2D: Type II diabetes
YFP: Yellow Fluorescent Protein
