# Supplementary figures and images for: Generation of an immortalized mesenchymal stem cell line producing a secreted biosensor protein for glucose monitoring
Source: PLoS One. 2017 Sep 26;12(9):e0185498. doi: 10.1371/journal.pone.0185498 (PMC5614622; doi:10.1371/journal.pone.0185498)

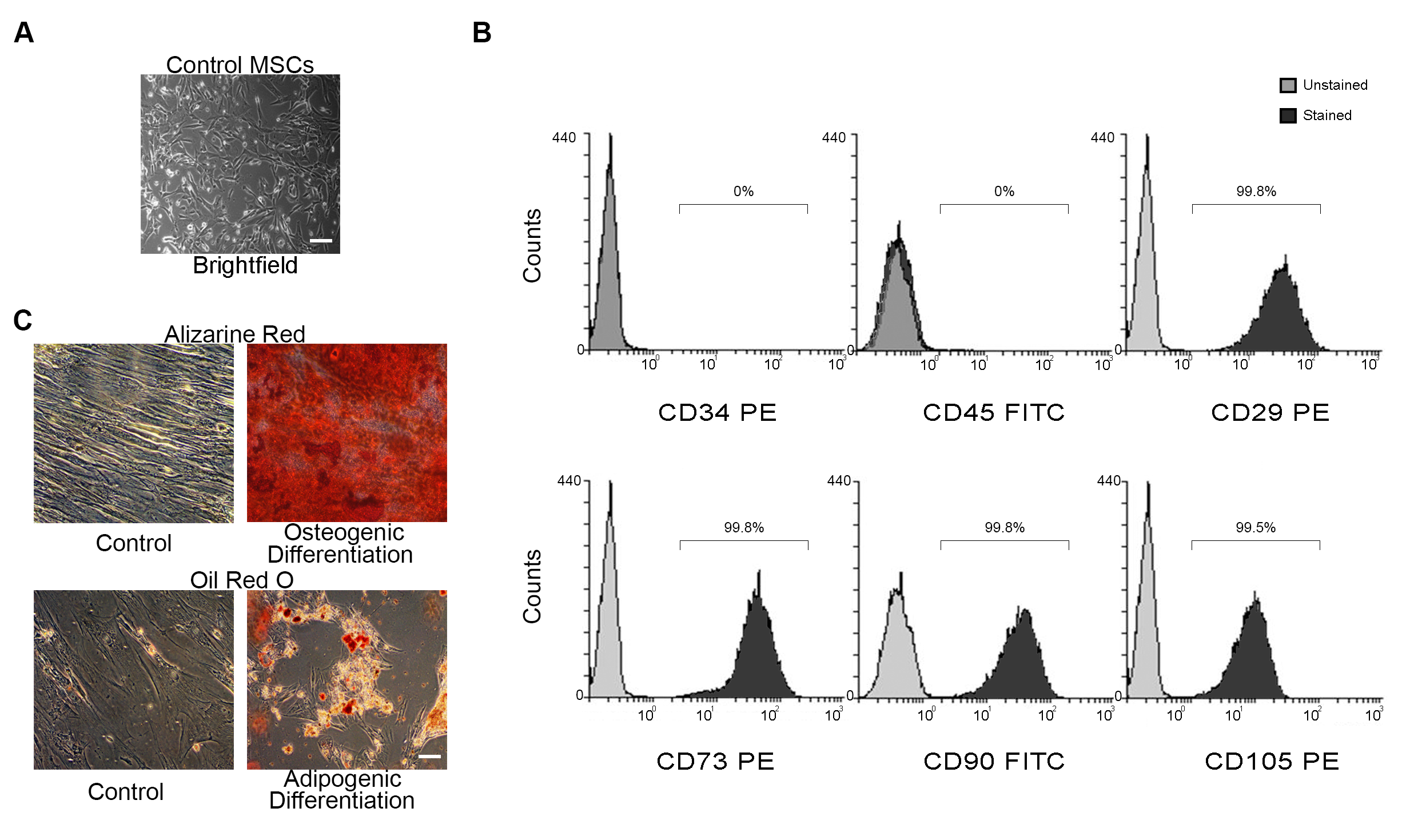

Supplement: S1 Fig — (A) Optical microscopy of control MSCs (scale bar: 100μm). (B) Flow cytometry for hemopoietic CD34, 45 or mesenchymal CD29, 73, 90, 105 markers. (C) Staining of control or differentiated cells with Alizarine Red/Oil Red O indicative of osteogenic/adipogenic differentiation, respectively (scale bar: 100μm). (TIF) [file pone.0185498.s001.tif]

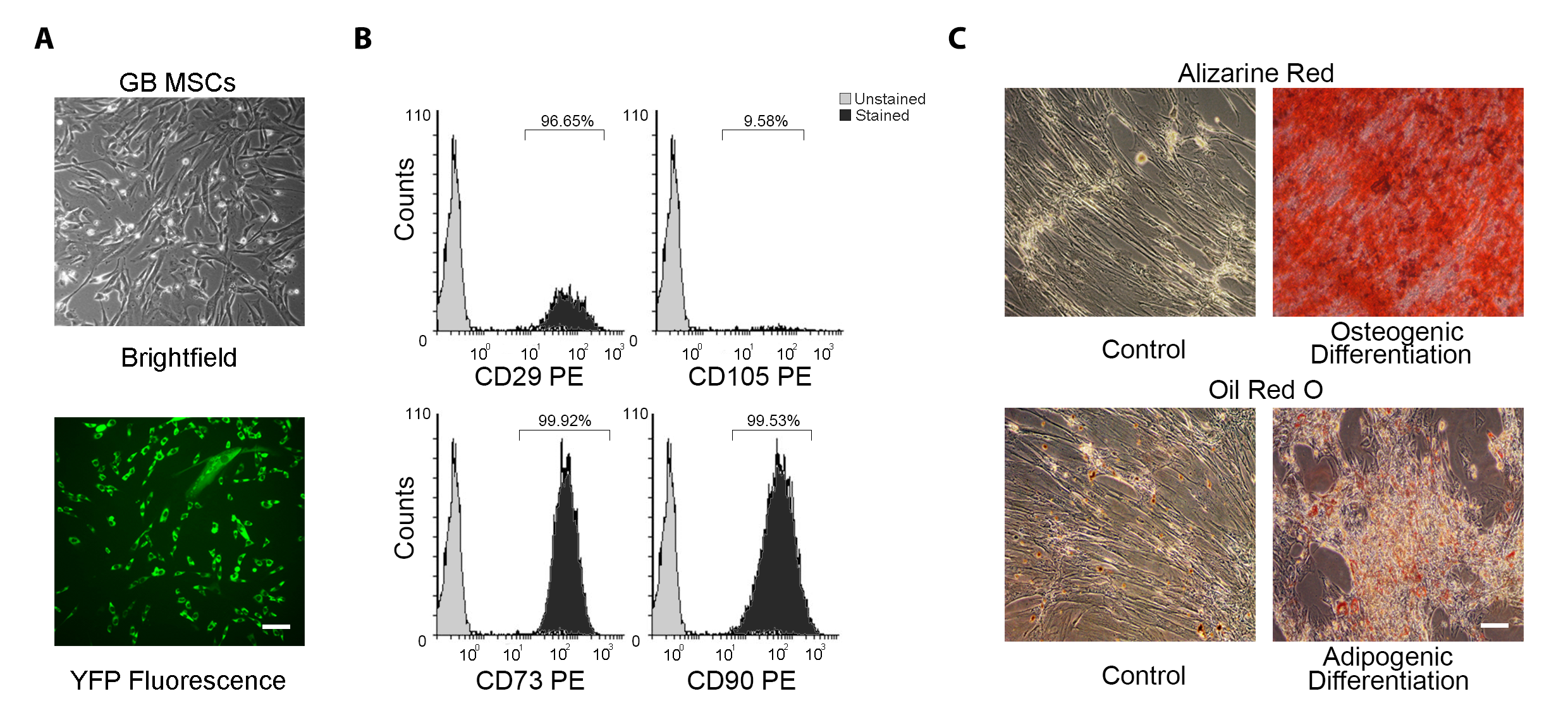

Supplement: S2 Fig — (A) Optical and fluorescence microscopy of GB MSCs (scale bar: 100μm). (B) Flow cytometry for mesenchymal CD29, 73, 90, 105 markers. (C) Staining of control or differentiated cells with Alizarine Red/Oil Red O indicative of osteogenic/adipogenic differentiation, respectively (scale bar: 100μm). (TIF) [file pone.0185498.s002.tif]

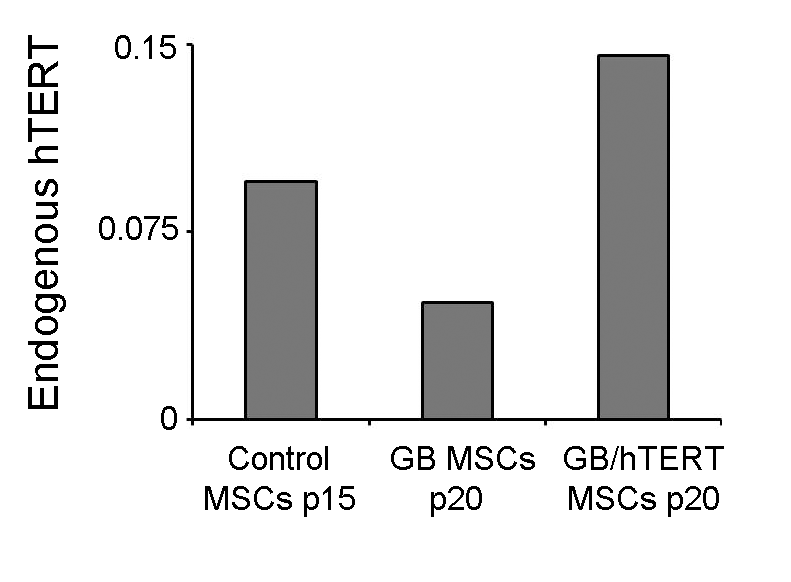

Supplement: S3 Fig — Protein levels of endogenous hTERT (Fig 3A) were quantified in control (passage 15), GB or GB/hTERT MSCs (passage 20) using the ImageJ software. GAPDH protein levels were used as loading controls. (TIF) [file pone.0185498.s003.tif]

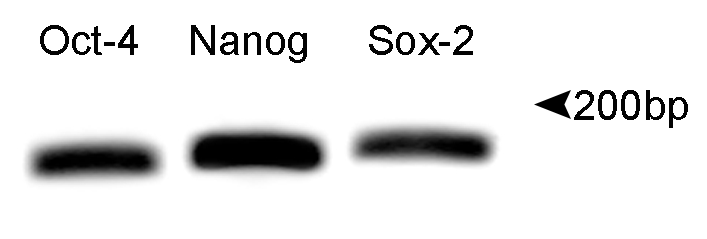

Supplement: S4 Fig — Amplified fragments of Oct-4, Nanog and Sox-2 mRNA in RT-qPCR reactions. (TIF) [file pone.0185498.s004.tif]

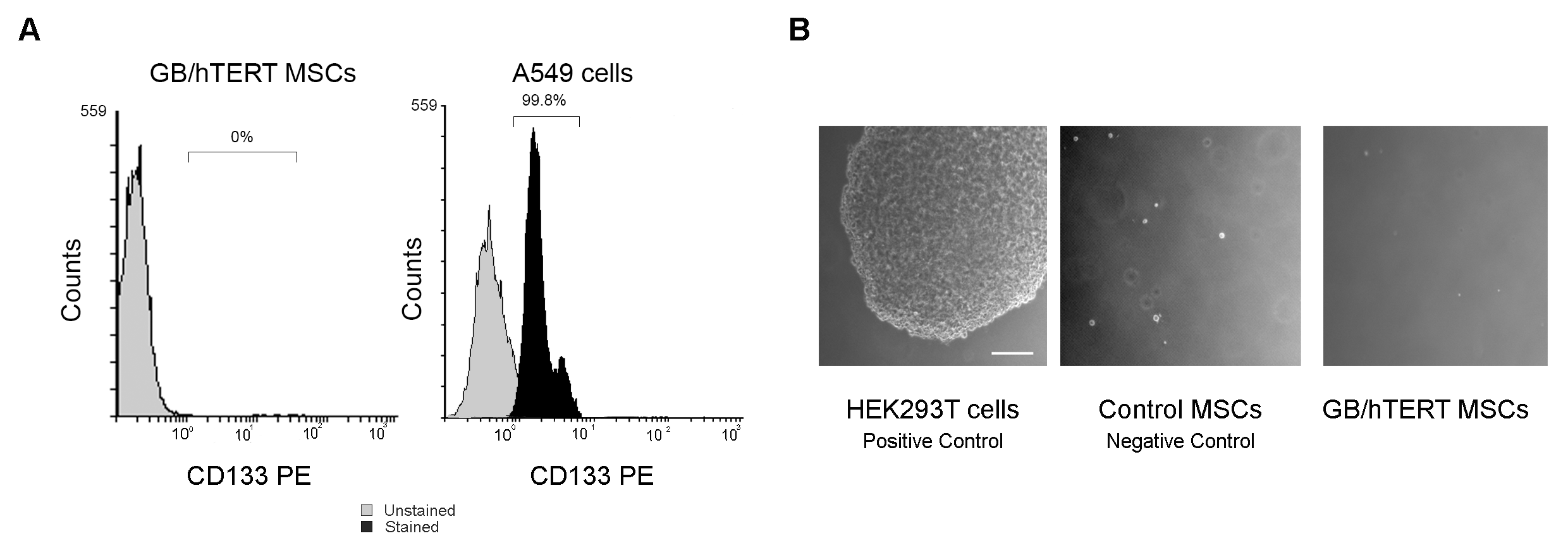

Supplement: S5 Fig — (A) Flow cytometry of GB/hTERT MSCs and lung cancer A549 cells for the cancer cell marker CD133. (B) Soft agar assays of GB/hTERT MSCs at passage 85. HEK293T cells and control MSCs at passage 15 were used as positive and negative control, respectively (scale bar: 50μm). (TIF) [file pone.0185498.s005.tif]

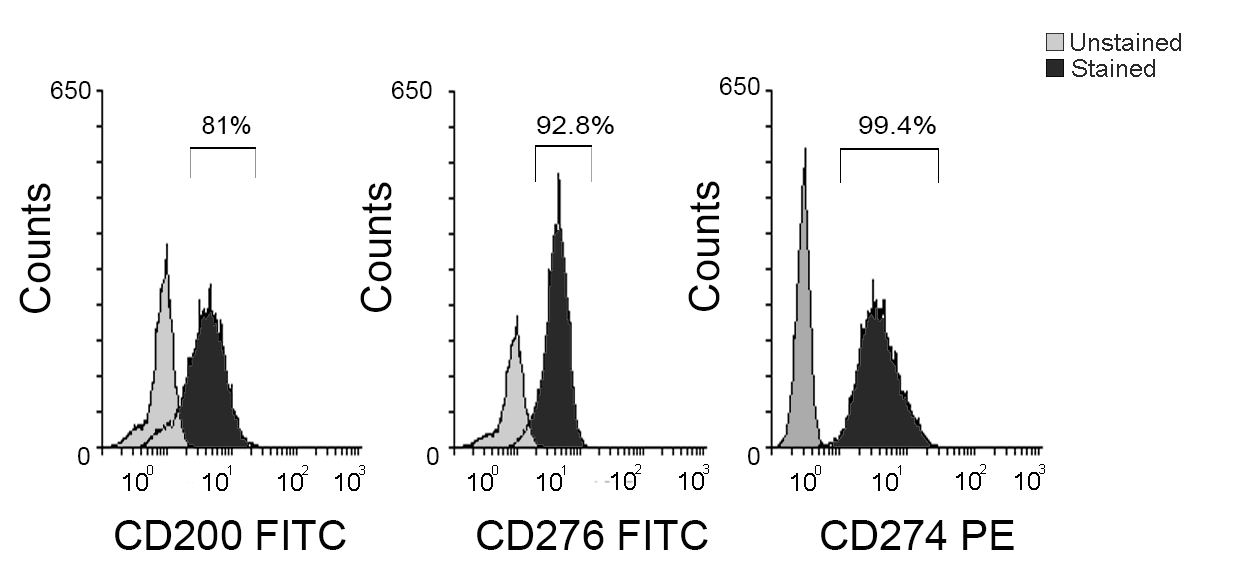

Supplement: S6 Fig — Flow cytometry of control MSCs for the immunomodulatory cell markers CD200, 276 and 274. (TIF) [file pone.0185498.s006.tif]

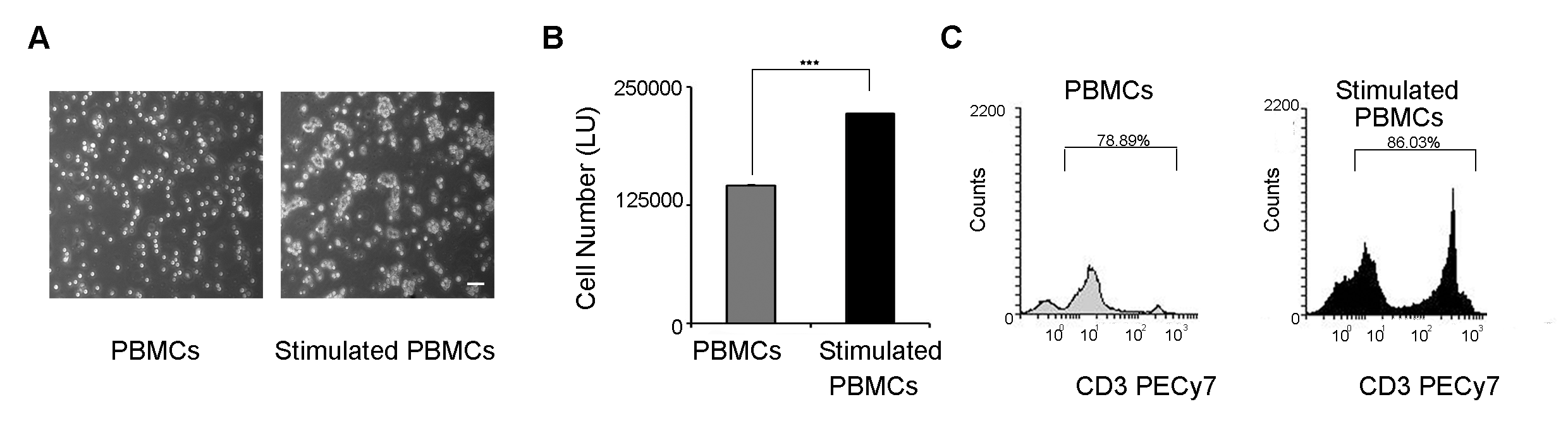

Supplement: S7 Fig — (A) Optical microscopy of unstimulated and stimulated PBMCs (scale bar: 100μm). (B) Measurement of cell proliferation. Luminescence units (LU) correspond to total cell number. Data from 3 independent experiments are presented as mean ± SD (***: p<0.001). (C) Flow cytometry of unstimulated and stimulated PBMCs for CD3 T cell marker. (TIF) [file pone.0185498.s007.tif]

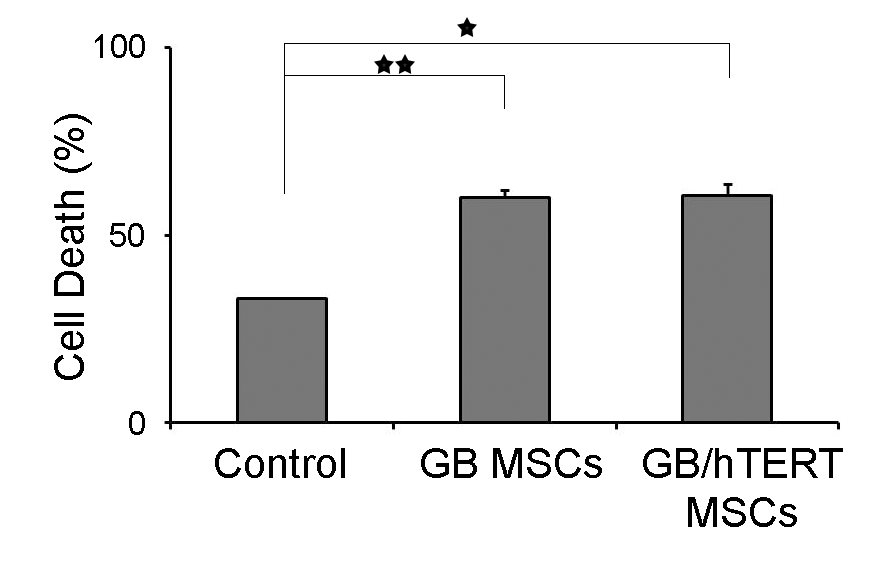

Supplement: S8 Fig — Cell death of stimulated PBMCs co-cultured with GB or GB/hTERT MSCs in transwell plates is expressed as a percentage (%) of total cell number. Control experiments were performed in the absence of MSCs in MLR assays. Data from 3 independent experiments are presented as mean ± SD (**: p<0.01, *: p<0.05). (TIF) [file pone.0185498.s008.tif]

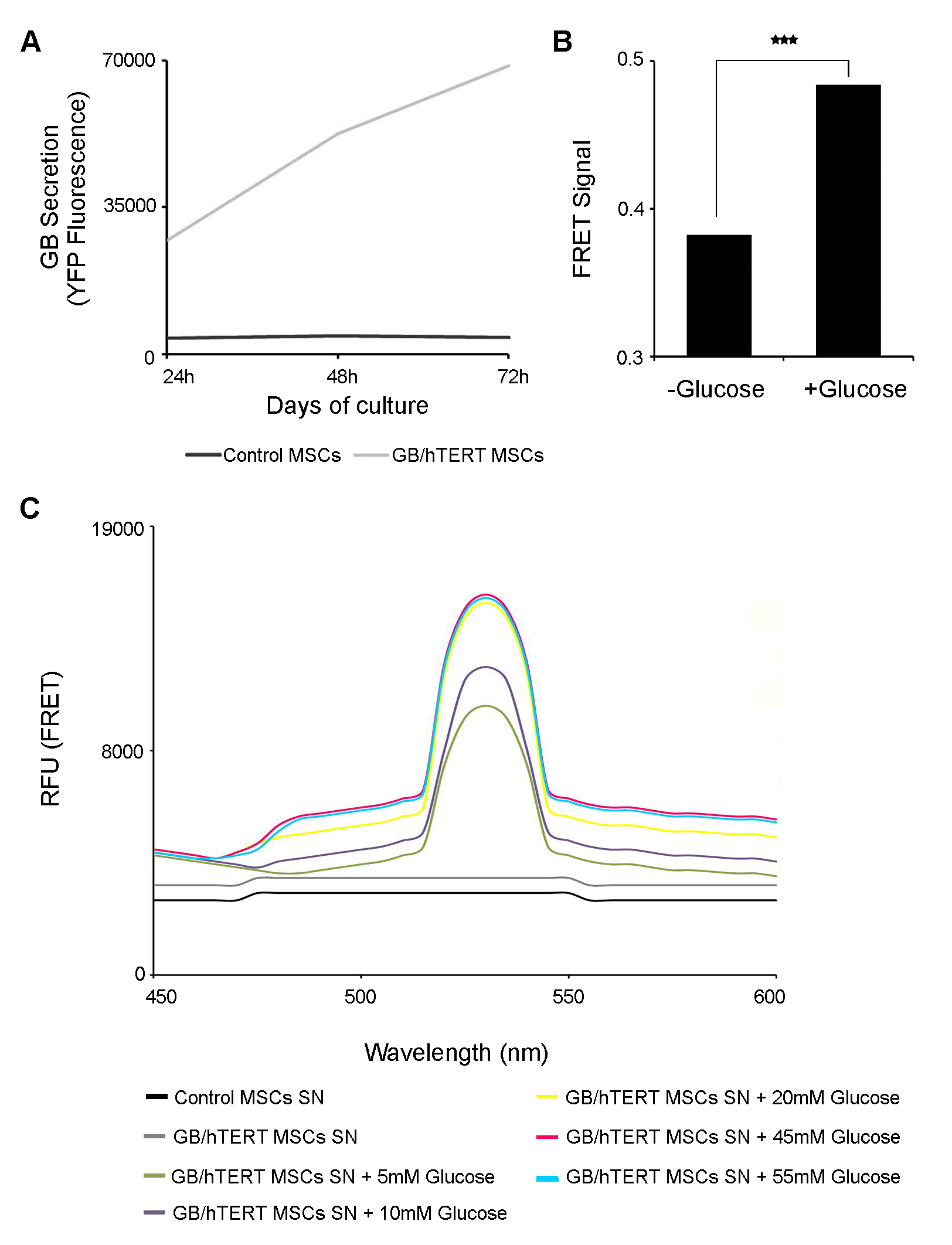

Supplement: S9 Fig — (A) Quantification of GB secretion by GB/hTERT MSCs as indicated by YFP measurement in cell culture supernatant (24-72h cultures). Control measurements were performed in samples from control MSC culture. (B) Detection of FRET signal in GB/hTERT cell culture supernatant in the absence or presence of glucose (25mM). Data from 3 independent experiments are presented as mean ± SD (***: p<0.001). (C) Fluorescence spectral scan analysis and detection of FRET signal in GB/hTERT cell culture supernatant mixed with various glucose concentrations (0-55mM) (RFU: Relative Fluorescence Units). (TIF) [file pone.0185498.s009.tif]

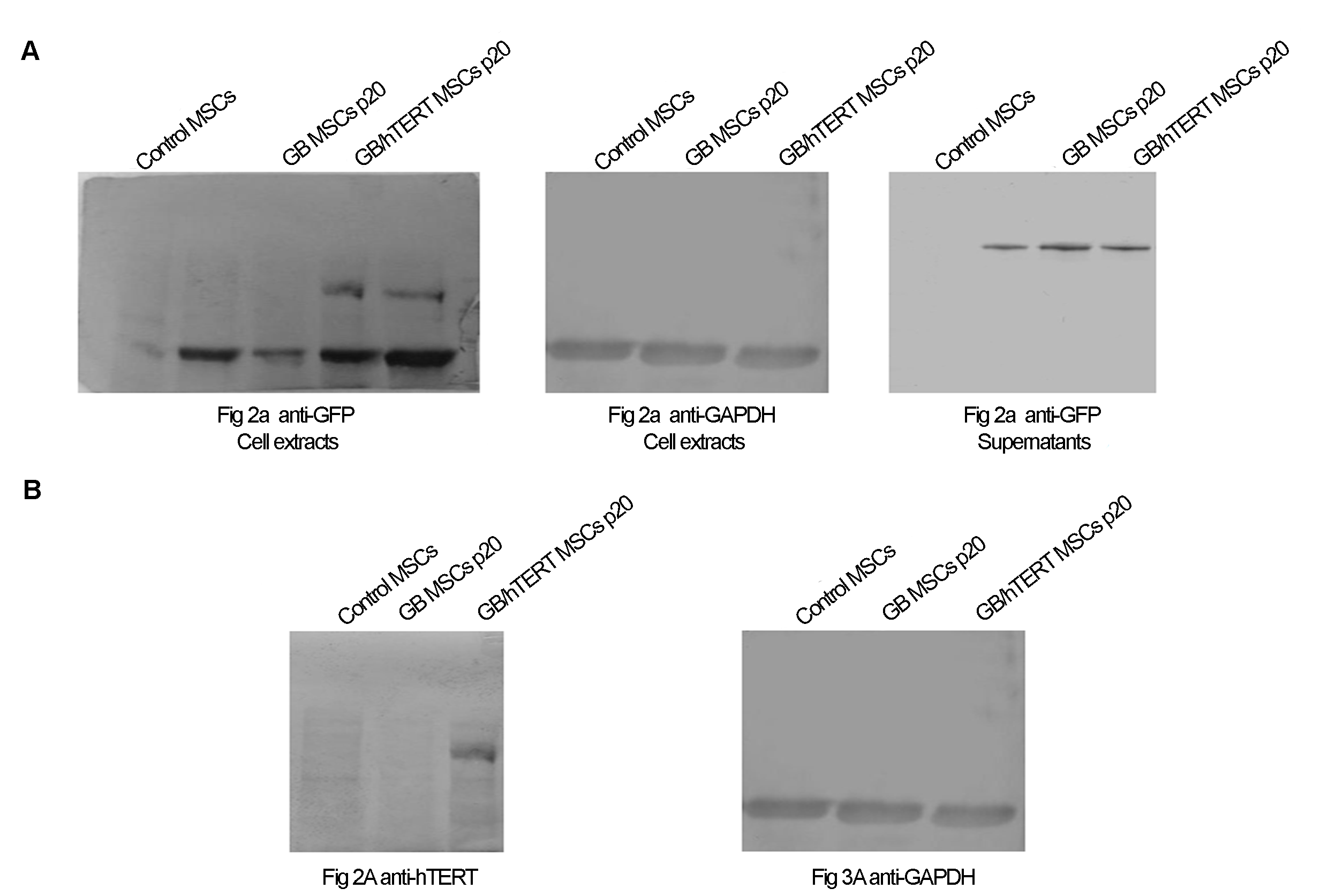

Supplement: S10 Fig — (TIF) [file pone.0185498.s010.tif]
